# Assessing Morphological Diversity of Acinic Cell Carcinoma of Salivary Glands at a Tertiary Care Hospital in Pakistan

**DOI:** 10.7759/cureus.63134

**Published:** 2024-06-25

**Authors:** Sahar Suleman, Saira Fatima, Nasir Ud Din

**Affiliations:** 1 Pathology and Laboratory Medicine/Histopathology, Aga Khan University Hospital, Karachi, PAK; 2 Histopathology, Aga Khan University, Karachi, PAK; 3 Pathology and Laboratory Medicine, Aga Khan University, Karachi, PAK

**Keywords:** papillary cystic, tubulocystic, follicular, solid, microcystic, de-differentiated, salivary gland tumor, acinic cell carcinoma

## Abstract

Introduction: Acinic cell carcinoma (AciCC) is a rare clinical entity and a salivary gland malignancy. It is associated with wide histological variations in the cytomorphological patterns.

Methods: Sixty cases diagnosed as AciCC from 2002 to 2023 were assessed for diverse cytomorphological patterns.

Results: The mean age of patients at the time of diagnosis was 44.35±16.8 years ranging from 15 to 81 years. Females comprised 58.3% for a F: M ratio of 1.4:1. Fifty three cases (88.3%) occurred in the parotid gland, two cases in the nasal region (3.3%), and one case each in the soft plate and upper lip (1.7%). The location of the remaining three cases was not specified. The most common presenting complaint was a well-defined facial swelling associated with pain. The average tumor size was 3.8±1.9 cm. The most predominant architectural pattern was solid (83.3%) followed by microcystic (60%), then follicular (41.7%), papillary cystic (14.3%), and tubulocystic (28.6%), and AciCC with de-differentiation/high-grade transformation was reported in three cases (5%). In 83.3% of the cases (50 out of 60), we noticed a mixture of two or more growth patterns. Other degenerative changes included prominent lymphoid stroma, hemorrhage, and cystic change.

Conclusion: Awareness and recognition of diverse cytomorphological patterns of AciCC, especially in institutions of a developing country where there is limited availability of highly specific and sensitive immunohistochemical stains or molecular diagnostics, are crucial and essential.

## Introduction

Salivary gland tumors are a heterogeneous group of neoplasms constituting about 3% to 10% of neoplasms of the maxillofacial region [1]. There were 53,583 newly reported cases of salivary gland tumors worldwide in 2020 and 22,778 tumor-related deaths [2]. Although currently a large-scale epidemiologic study does not exist catering to the incidence of salivary gland tumors nationwide, a comprehensive single-center study conducted over five years in Pakistan showed an overall frequency of salivary gland cancers among malignancies of the general body to be 0.8% and within head and neck tumors to be 6.9% [3]. Benign tumors, particularly pleomorphic adenomas, were more common as compared to malignant ones. Mucoepidermoid carcinoma was the common malignancy followed by adenoid cystic carcinoma [3].

Acinic cell carcinoma (AciCC) accounts for 10% of all salivary gland malignancies, up to 18.7% of the cases arising in the parotid gland [4]. The World Health Organization (WHO) currently defines salivary AciCC as ‘‘a malignant epithelial neoplasm of salivary glands in which at least some of the neoplastic cells demonstrate serous acinar cell differentiation, characterized by cytoplasmic zymogen secretory granules. Salivary ductal cells are also a component of this neoplasm’’ [5]. An extremely unusual variant of this tumor has also been described as “Acinic cell carcinoma with high-grade transformation,” associated with aggressive behavior and a poor prognosis contrary to its traditional counterpart [6]. Morphologically, AciCC closely resembles the normal serous acinar cells of salivary gland parenchyma, but the coexistence of other cell types such as intercalated, vacuolated, ductal, and clear types provides wide histological variations in the cytomorphological patterns of salivary gland AciCC. Over the years, the authors have adapted to the allotted descriptive categories (solid, microcystic, papillary-cystic, tubulo-cystic, and follicular) which were first presented by Abrams et al. in 1965 in a detailed clinicopathologic study of 77 cases [7]. These categories have been useful to general pathologists worldwide and are still relevant today.

The morphological diversity also results in overlapping features with other benign and malignant salivary gland neoplasms, often leading to diagnostic difficulty and interobserver variation. In this context, the use of immunohistochemistry, ancillary studies, and molecular analysis for genomic rearrangements are essential. Haller et al. demonstrated that AciCC harbors a t(4;9)(q13;q31) rearrangement that causes the active enhancer regions of the secretory Ca-binding phosphoprotein (SCPP) gene cluster upstream of the NR4A3/NOR-1 gene, resulting in the upregulation of NR4A3 via enhancer hijacking and an early oncogenic event in the pathogenesis [8].

Immunohistochemical stain DOG-1 is a marker of acinar differentiation and is selectively expressed in the luminal plasmalemma of serous and intercalated ductal cells [9]. Results of initial studies showed its promising utility for the diagnosis of AciCC. However, subsequent studies showed that it is also frequently positive in adenoid cystic carcinoma, epithelial-myoepithelial carcinoma, pleomorphic adenoma, polymorphous low-grade adenocarcinoma, and others [9]. Recently, Skaugen et al., in their study, evaluated the diagnostic utility of NR4A3 IHC and concluded that it is highly specific (100%) for AciCC and more sensitive (81.8%) than DOG-1 [10].

Treatment involves complete surgical resection. Incomplete excision portends a poor prognosis. Radiotherapy is not usually required for initial disease management unless adverse factors are present, including positive surgical margins, perineural invasion, and cervical lymph node involvement. Overall survival rates approach 97%, 93%, and 89% at 5, 10, and 15 years, respectively [11].

In our general pathology practice, instances always arise when a pathologist gets stuck in a perplexed situation deciphering the type of salivary gland tumor due to overlapping cytomorphology and acquiring consultation. Another setback in our resource-limited setting is the unavailability of various immunohistochemical markers and testing for molecular rearrangements. Therefore, histological examination is considered the gold standard, and an understanding and recognition of the morphological patterns of AciCC are emphasized among pathologists. 

This study aims to examine, assess, and analyze clinicopathologic features and compare any key similarities and differences of AciCC with studies reported in other parts of the world. AciCC is a rare salivary gland neoplasm, and there isn't much regional database available to us at present.

## Materials and methods

Study design and exclusion and inclusion criteria

This is a descriptive observational study. Our histopathology lab is one of the largest referral centers in the country, and we receive close to 500, either incisional biopsies or resected specimens of salivary gland tumors annually from all over the country. All the cases diagnosed as AciCC from 2002 to 2023 were searched electronically in the institutional Integrated Laboratory Management System (ILMS). Autolyzed, poorly fixed samples were disregarded, along with samples of patients with a prior history of radiotherapy.

Data collection

Approval from the hospital ethical research committee was sought (2023-7495-27326). Most of the cases in our cohort were referrals. Patient information such as demographics including age, gender, presenting complaints, duration of symptoms, tumor site, size, and surgical procedure was extracted from the histopathology reports. H&E and immunostained slides were retrieved and reviewed by two pathologists having a special interest in head and neck pathology. A record in the form of proforma was made, which included morphological patterns, concurrent lymph node involvement, and perineural invasion. Additional histologic findings such as tumor necrosis, lymphoid stroma, hemorrhage, and cystic degeneration were also noted. Ancillary studies, including immunohistochemical and special stains, were reviewed and tumor staging was conducted according to the American Joint Committee on Cancer (AJCC) system.

Statistical analysis

Descriptive statistical analysis was performed using Stata version 17 (StataCorp LLC, College Station, USA). Frequencies and percentages were obtained for all the categorical variables including gender, tumor site, morphological patterns and pathologic T stage. Measures of central tendency and variability were obtained for continuous data such as age and tumor size. 

## Results

A total of 65 cases of AciCC were retrieved. Upon reviewing the slides, five cases of AciCC were found to be suspicious for secretory carcinoma and were evaluated for ETV6 gene rearrangement using a break-apart ETV6 probe by FISH. These cases were included in our other study. The final cohort consisted of 60 cases diagnosed as AciCC based on a histological review of slides. The mean age of patients at the time of diagnosis was 44.35±16.8 years, ranging from 15-81 years (Table 1). There were 50 (83.3%) resection specimens and 10 (16.7%) were incisional biopsies. Additionally, selective cervical lymph node sampling of three patients (5%) was received with the initial biopsy specimen. The average tumor size was 3.8±1.9 cm. Females comprised 58.3% of cases, resulting in a female-to-male ratio of 1.4:1. A total of 53 cases (88.3%) occurred in the parotid gland, with two cases (3.3%) in the nasal region, and one case (1.7% each) each in the soft palate and upper lip. The location of the remaining three cases (5%) was not specified by the referring physician (see Table 1). The most common presenting complaint was a well-defined facial swelling associated with pain. Out of the cases, 50 (83.3%) were resection specimens and 10 (16.7%) were incisional biopsies.

**Table 1:** Summary of clinicopathological features (n=60). *According to the AJCC guidelines. AJCC: American Joint Committee on Cancer

Clinicopathological features	Frequency (%)	p-value (≤0.05)
Age (years)		
Range	15-81	0.33
Mean	44.35±16.8	
Median	45	
Gender (n=60)		
Male	25 (41.7%)	-
Female	35 (58.3%)	
Site (n=60)		
Parotid gland	53 (88.3%)	-
Nasal	2 (3.3%)	
Soft palate	1 (1.7%)	
Upper lip	1 (1.7%)	
Not known	3 (5.0%)	
Size (cm)		
Range	0.5-10	0.90
Mean	3.8±1.9	
Median	3.5	
Morphological features (n=60)		
Solid	50 (83.3%)	-
Microcystic	36 (60.0%)	
Follicular	25 (41.7%)	
Papillary cystic	7 (14.3%)	
Tubulocystic	14 (28.6%)	
De-differentiation/High-grade transformation	3 (5%)	
Pathologic T stage * (n=60)		
pT1	4 (6.7%)	-
pT2	36 (60%)	
pT3	20 (33.3%)	

**Figure 1:**
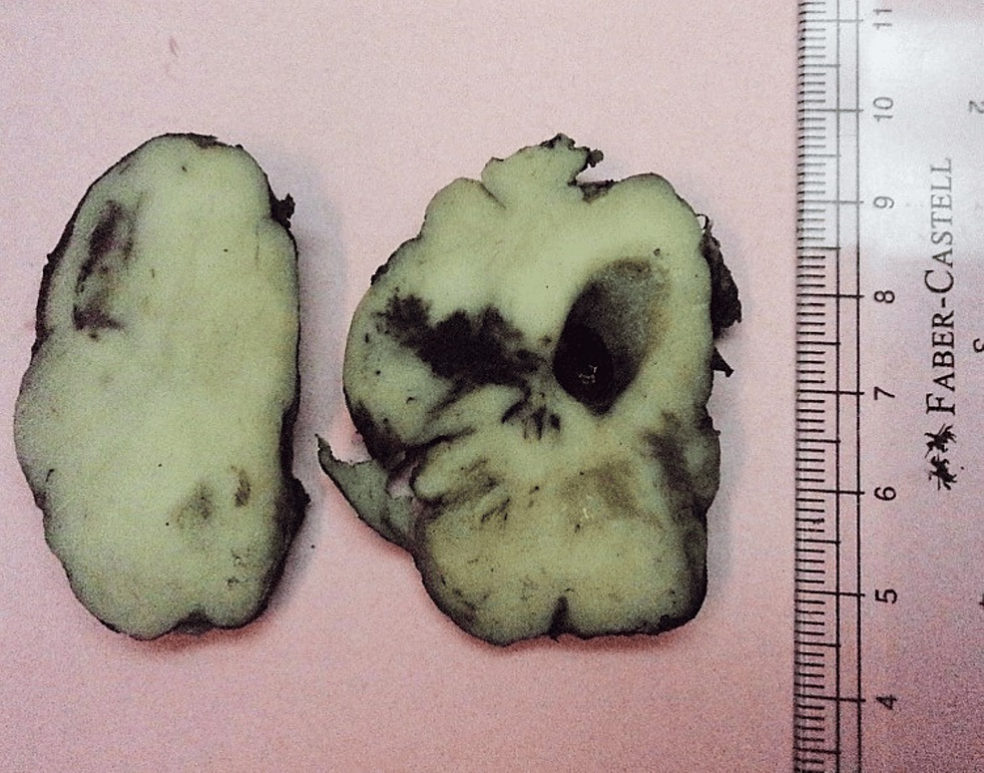
Photomicrograph of a parotid gland involving AciCC. Macroscopic appearance shows salivary gland tissue completely involving a tan-white homogenous lesion with cystic degenerative, hemorrhagic changes. The tumor involves the inked resection margin. AciCC: Acinic cell carcinoma

Macroscopic appearance showed a homogeneous lesion involving the salivary gland with cystic and hemorrhagic changes (Figure 1).

**Figure 2:**
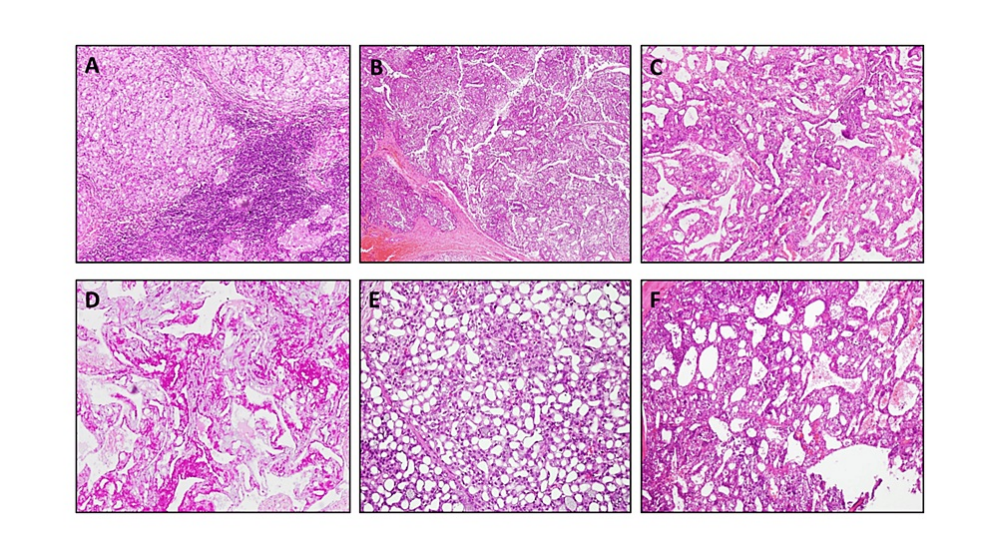
Morphological patterns of AciCC. A: AciCC exhibiting a solid lobulated architectural pattern with prominent lymphoid stroma (H&Ex20). B: Tubulocystic architectural pattern (H&Ex20). C: Papillary cystic architectural pattern exhibiting papillary projections with delicate fibrous cores (H&Ex20). D: Special stain periodic acid Schiff alcian blue, with diastase, highlighting intracytoplasmic granules (x20). E: Microcystic architectural pattern exhibiting cystic lumina (H&Ex20). F: A macrofollicular architectural pattern is characterized by cystic spaces filled with eosinophilic secretions (H&Ex20). AciCC: Acinic cell carcinoma

**Figure 3:**
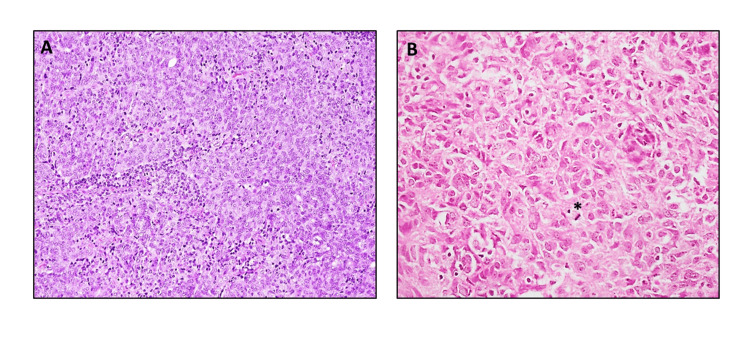
AciCC with de-differentiation/high-grade transformation. A & B: Tumor show diffuse sheets of neoplastic cells exhibiting anaplasia, nuclear pleomorphism, increased nuclear to cytoplasmic ratio, and prominent mitoses (*) (H&Ex20). AciCC: Acinic cell carcinoma

**Figure 4:**
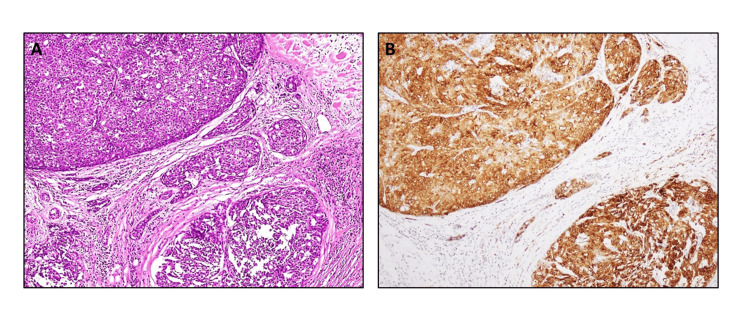
Application of DOG-1 immunohistochemical stain. A : Solid, multilobulated architectural pattern of AciCC (H&Ex20). B: DOG-1 immunohistochemical stain, exhibiting positive cytoplasmic and membranous expression (x20). AciCC: Acinic cell carcinoma

The different morphological patterns of AciCC with the application of special and immunohistochemical stains are shown in Figures 2A-2F, 3A, 3B, 4A, 4B.

Twenty-seven cases (45%) in our cohort demonstrated a prominent accompanying lymphoid stroma, with occasional germinal centers. Other degenerative changes, including tumor necrosis, hemorrhage, and cystic change, were observed. Perineural invasion was noted in only three cases (5%).

Ancillary testing included periodic acid Schiff diastase resistant and alcian blue/mucicarmine, both of which detected zymogen granules. Immunohistochemical stains included keratin 7, DOG1, P63, and S100, performed either individually or within a panel.

Follow-up was available for 44 patients (73.3%) in our cohort. The follow-up period ranged from 6 months to up to 12 years. Twenty-five patients (56.8%) were alive with no residual or metastatic disease. Five patients (11.3%) had died due to metastatic disease or recurrence.

## Discussion

A recent analysis of Surveillance, Epidemiology, and End results (SEER) data from 1973 to 2009 indicated that AciCC has a higher average annual incidence among females (0.15 cases per 100,000 patients) compared to males (0.11 cases per 100,000 patients) [4]. This finding correlates with a consistent slight female predominance in our institutional series. Most of the patients in the current series, presented during their fifth decade of life, with a median age of 44.3 years, similar to the average of 45.75 years reported by Munteanu et al. [12], and 45 years (over 60% of patients) reported by Neskey et al. [13].

The usual clinical presentation is a slow-growing swelling with intermittent pain, which patients often ignore, resulting in a late diagnosis. Nodal metastasis is infrequent at presentation. In our series, 3 out of 60 (5%) patients underwent concurrent, selective cervical lymph node sampling, but there was no lymph node metastasis in any of these cases. In contrast, a case series from the MD Anderson Cancer Center reported 12 out of 155 cases (8 %) with nodal disease [13], while another series from the Memorial Sloan Kettering Cancer Center (MSKCC) found simultaneous nodal disease in 3 out of 35 patients (9%) at presentation [14]. Additionally, in a series by van Weert et al., nodal disease was observed in 2 out of 89 cases (2%) [15]. In our series, 88.3% of the tumors were located in the parotid gland, similar to the findings of van Weert et al., where 85% of the tumors were found in the parotid gland [15].

In our study, the most predominant architectural pattern was solid in 50 cases (83.3%), followed by microcystic in 36 cases (60%), follicular in 25 cases (41.7%), papillary cystic in seven cases (14.3%), tubulocystic in 14 cases (28.6%), and AciCC with de-differentiation/high-grade transformation, which was confirmed in three cases (5%) (Table 1). Additionally, a mixture of two or more growth patterns was observed in 50 cases (83.3%).

We observed that the solid pattern was characterized by a lobulated architecture composed of sheets and aggregates of serous cells separated by fibrous septae. Cystic lumina was also present within the solid lesions, giving them a microcystic appearance. In some areas, the cysts appeared to be coalesced and represented ruptured or degenerated vacuolated cells.

The papillary cystic pattern consisted of papillary projections with delicate fibrous cores, and in some areas, pseudo-papillae accompanied by macro and micro cysts. The luminal epithelial cells exhibited a hobnail appearance.

The follicular pattern was characterized by epithelial-lined cystic spaces filled with eosinophilic secretions. The individual serous acinar cells were large and polygonal in shape, with abundant slightly basophilic cytoplasm containing dense, coarse zymogen granules. Vacuolated cells exhibited clear cytoplasmic vacuoles, and intercalated duct-type cells surrounded luminal spaces. These duct cells were cuboidal and had centrally located nuclei.

Tumors re-classified as Secretory carcinoma exhibited papillary cystic, microcystic, and follicular appearances, with cells containing secretions positive for periodic acid-Schiff diastase [16].

Three cases (5%) were distinguished from conventional AciCC by comparing morphological features studied and illustrated by Skálová et al, in their study of nine cases of AciCC with high-grade transformation [17]. These histological features included anaplasia, nuclear pleomorphism, increased nuclear to cytoplasmic ratio, mitoses of >2/10HPF, a higher proliferative index (Ki- 67 ⁄ MIB-1 > 5%), and geographical necrosis. Additionally, all three cases demonstrated a low-grade component with solid and microcystic growth patterns, leading to their categorization as ‘De-differentiated’ or, ‘AciCC with high-grade transformation’.

Perineural invasion is a rare feature in AciCC, as demonstrated in our cohort with an incidence of 5% (3 cases), compared to 8% in the Amsterdam case series [15], and an even higher incidence of 23% reported by Gomez et al. [14]. Two of these cases were AciCC with high-grade transformation. Despite its relatively low incidence, its presence has a negative impact on survival, as is observed in other salivary gland carcinomas [14].

Histological diagnosis was aided by assessing the expression of special stain periodic acid-Schiff with or without diastase digestion in 28 cases (46.7%), wherein intracytoplasmic granules were highlighted in all.

Immunohistochemical stain DOG-1 was performed in 19 cases (31.7%), since its procurement in our institution in 2017, revealing an admixture of membranous and cytoplasmic expression in all cases. Keratin 7 was performed in 13 cases (21.7%) and showed positive membranous expression, while P63 had negative expression in the six cases (10%) tested, and S100 had negative expression in the four cases (6.7%) tested. In the literature, the diagnostic utility of MUC4 has been evaluated for Secretory carcinoma and found to be a robust marker. Therefore, in problematic cases, it can be used as an adjunctive tool [18]. Positive expression of MUC4 helped us distinguish and exclude five cases of secretory carcinoma from AciCC.

In our cohort, tumors were classified as pT1 in four cases (6.7%), pT2 in 36 cases (60%), and pT3 in 20 cases (33.3%), whereas the pathologic N stage was pN0 in all three cases (5%) with concurrent cervical lymph node sampling.

Among the five (8.3%) deceased patients, three had metastatic disease. Metastases were found in the lungs in two cases and the cerebellopontine angle in one case. One of the deceased patients was diagnosed with AciCC with high-grade transformation; however, the cause of death was not entirely disease-related.

The limitations of our study included a small sample size, poor antibody retrieval techniques, and limited availability of follow-up.

## Conclusions

AciCC exhibits diverse morphological patterns that overlap with those of other salivary gland neoplasms, a finding consistent with international published data. Therefore, familiarity with and recognition of these diverse features, particularly in institutions in developing countries where highly specific and sensitive immunohistochemical stains or molecular diagnostics may be limited, are crucial.

In our study, we elaborate on the pertinent histomorphological features of the various patterns of AciCC, which we found helpful in differentiating AciCC from other salivary gland tumors, especially secretory carcinoma. Furthermore, this study contributes to the limited regional database available for future reference.
